# The *Drosophila* functional Smad suppressing element *fuss*, a homologue of the human *Skor* genes, retains pro-oncogenic properties of the Ski/Sno family

**DOI:** 10.1371/journal.pone.0262360

**Published:** 2022-01-14

**Authors:** Mathias Rass, Laura Gizler, Florian Bayersdorfer, Christoph Irlbeck, Matthias Schramm, Stephan Schneuwly

**Affiliations:** Department of Developmental Biology, Institute of Zoology, University of Regensburg, Regensburg, Germany; California State University Los Angeles, UNITED STATES

## Abstract

Over the years Ski and Sno have been found to be involved in cancer progression e.g. in oesophageal squamous cell carcinoma, melanoma, oestrogen receptor-positive breast carcinoma, colorectal carcinoma, and leukaemia. Often, their prooncogenic features have been linked to their ability of inhibiting the anti-proliferative action of TGF-ß signalling. Recently, not only pro-oncogenic but also anti-oncogenic functions of Ski/Sno proteins have been revealed. Besides Ski and Sno, which are ubiquitously expressed other members of Ski/Sno proteins exist which show highly specific neuronal expression, the SKI Family Transcriptional Corepressors (Skor). Among others Skor1 and Skor2 are involved in the development of Purkinje neurons and a mutation of Skor1 has been found to be associated with restless legs syndrome. But neither Skor1 nor Skor2 have been reported to be involved in cancer progression. Using overexpression studies in the *Drosophila* eye imaginal disc, we analysed if the *Drosophila* Skor homologue Fuss has retained the potential to inhibit differentiation and induce increased proliferation. Fuss expressed in cells posterior to the morphogenetic furrow, impairs photoreceptor axon pathfinding and inhibits differentiation of accessory cells. However, if its expression is induced prior to eye differentiation, Fuss might inhibit the differentiating function of Dpp signalling and might maintain proliferative action of Wg signalling, which is reminiscent of the Ski/Sno protein function in cancer.

## Introduction

Transforming growth factor beta (TGF-ß) signalling is involved in a wide range of processes during development e.g. cell adhesion, bone morphogenesis and cell motility [1]. Upon binding of a ligand of the TGF-ß superfamily to a Type II receptor, the Type II receptor recruits a Type I receptor and activates the Type I receptor by phosphorylation. Then, the Type I receptor phosphorylates receptor regulated smads (R-Smads), which then can bind to the common mediator Smad SMAD4 and translocate as a R-Smad/Smad4 complex into the nucleus, where these complexes interact with co-activators to activate gene expression [1]. Negative regulators of the TGF-ß signalling pathway are inhibitory Smads (I-Smads), Smurfs and the Ski/Sno protein family [2–4]. Proteins of the latter group possess two structural domains: the Ski/Sno homology domain and the SMAD4-binding domain [5, 6]. With the help of these domains, Ski/Sno proteins can interact, among others, with R-Smads, N-CoR, Sin3a, SMAD4 and the histone deacetylase HDAC1 and this complex leads to transcriptional repression of target genes [7–11]. By their expression domains, Ski/Sno proteins can be further subdivided into ubiquitously expressed genes (human Ski and Sno), and mainly neuronally expressed genes, the SKI Family Transcriptional Corepressors (Skor1 and Skor2 [8–11]). The Ski/Sno proteins fulfil a wide range of different physiological functions such as axonal morphogenesis [12], Purkinje cell development [13], myogenesis [14] and mammary gland alveogenesis [15].

However, the Ski/Sno proteins were not discovered by their physiological functions but via the transforming capability of the viral ski (v-ski) homologue found in the Sloan-Kettering virus [16]. The first evidence that Ski/Sno proteins possess oncogenic capabilities came from overexpression experiments, where it was shown that not the truncation of v-ski is responsible for the transformation of chicken embryo fibroblasts, but that overexpression of v-ski, Ski or Sno is sufficient for this transformation [17]. Despite this background, their role in carcinogenesis is still not fully understood, if not even contradictory at times. Ski and Sno have been found to be upregulated in different types of cancer e.g. oesophagus squamous cell carcinoma [18], melanoma [19], and colorectal cancer [20]. Further evidence for a pro-oncogenic role was found in downregulation analyses of Sno or Ski. This downregulation resulted in decreased tumour growth in breast cancer cells [21] and pancreatic cancer cells [22]. But as stated before, there is some objection that Ski and Sno function purely as oncogenes. Mice, which were heterozygous mutant for Ski or Sno, showed an increased level of tumour induction after carcinogen treatment [23, 24]. In metastatic non-small cell lung cancer, Ski expression is significantly reduced, whereas increased expression of Ski in these cells reduced the invasiveness inhibiting epithelial-mesenchymal transition [25]. Therefore, this could reflect that the outcome of Ski or Sno expression in cancer cells is dependent on the cell type or the actual status of the cancer cells and cancer cells often exploit Ski or Sno to inhibit the anti-proliferative effects of TGF-ß signalling. Whereas Ski or Sno have been found to be involved in a lot of different cancer types, there is sparse evidence for deregulation of Skor proteins in cancer cells. Endogenously, Skor proteins have been linked to neurodevelopmental processes. After Skor1 overexpression, genes involved in axonal guidance or post-synapse assembly were differentially expressed [26]. Skor2 is important for cerebellar Purkinje cell differentiation as in Skor2 knockout mice dendrite formation of Purkinje cells was impaired [9, 13]. Pathophysiologically, Skor1 has mainly been linked to restless leg syndrome [26] and localized scleroderma [27].

In *Drosophila melanogaster*, only one homologue of Ski and Sno, which is designated Snoo [28], and one homologue of Skor1 and Skor2, which is designated Fuss, exist [4, 29]. We have recently shown that Fuss is interacting with SMAD4 [4] and HDAC1 [30]. In overexpression assays, Fuss can inhibit Dpp signalling [4, 29] and endogenously, the Fuss/HDAC1 complex is required for bitter gustatory neuron differentiation [30] and *fuss* mutant flies pause more often during walking [31]. However, we were interested if the Skor/Fuss proteins retained their ability to inhibit differentiation and induce increased proliferation. For this purpose, we overexpressed Fuss in differentiating cells of the eye imaginal disc, an excellent model tissue to study regulatory gene function in the context of carcinogenesis [32–34]. This overexpression impaired photoreceptor axon guidance and inhibited the differentiation of accessory cells such as cone cells and primary pigment cells, which are all transformed into a basal pigment cell type. In a second approach we generated *fuss* overexpressing clones early during development in the eye imaginal discs, when cells are still proliferating. This resulted in vast outgrowths of undifferentiated tissue of the eye imaginal disc because *fuss* overexpression most likely inhibited Dpp-signalling, a member of the TGF-ß superfamily. Our work shows that Fuss retained the ability of Ski/Sno proteins to inhibit the antiproliferative effects of TGF-ß signalling by analogous inhibition of Dpp-signalling, allowing proliferation to be sustained.

## Results

### *fuss* overexpression leads to a smooth eye surface and impairs axonal pathfinding

As Ski/Sno proteins have been found to be involved in cancer development and progression, we were interested if *fuss* overexpression can also inhibit differentiation and induce increased proliferation and hyperplasia, respectively. To answer this question, we chose the eye imaginal disc as a model tissue, because *fuss* is not endogenously expressed in the eye imaginal disc [30]. Furthermore, Fuss and its homologues are negative regulators of BMP/Dpp signalling in overexpression assays and consequently the eye imaginal disc enables us to investigate *fuss* overexpression in a Dpp independent and dependent context. First, we overexpressed *fuss* via the GMR-GAL4 driver line. GMR-GAL4 is active posterior to the morphogenetic furrow [35], which is the source of the Dpp morphogen during eye development [36] and thus Fuss cannot directly interfere with Dpp signalling.

**Fig 1:**
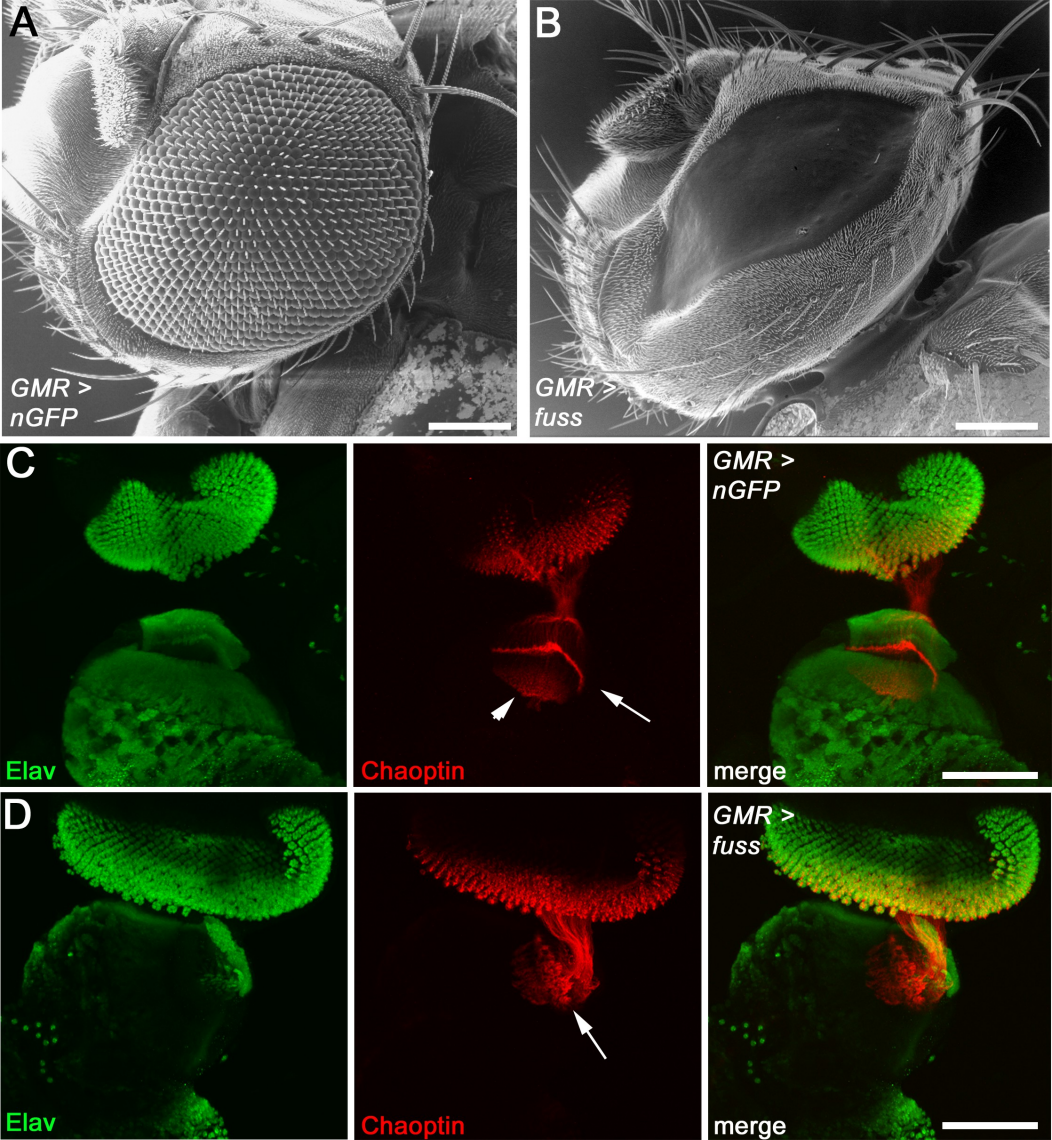
*fuss* overexpression leads to a smooth eye surface and impairs axonal pathfinding. (A) Eye from control flies (GMR-GAL4 > UAS-*nGFP*). (B) Eyes from *fuss* overexpressing flies (GMR-GAL4 > UAS-*fuss*). (C) Developing photoreceptors from control flies express Elav and Chaoptin. R1-R6 photoreceptor axons are terminating in the lamina plexus (arrows), whereas R7 and R8 photoreceptor axons are terminating in the developing medulla (arrowhead). (D) Photoreceptors overexpressing *fuss* are expressing Elav and Chaoptin, but photoreceptor axons are impaired in targeting accurately the lamina plexus and the medulla (arrows). (C, D) Confocal images are Z-stacks. Scale bars: 100 μm.

Interestingly, in adult *Drosophila* flies, where *fuss* was overexpressed with GMR-GAL4 during eye development, this overexpression leads to massive differentiation defects exhibiting a smooth, red coloured eye surface devoid of any typical eye structures such as ommatidia or bristles and we observed little to no phenotypic variability in these flies (Fig 1A and 1B). To see if photoreceptor induction is also affected by *fuss* overexpression, eye imaginal discs of late third instar larvae were stained with antibodies against Elav, a marker for neurons [37] and Chaoptin, a marker for photoreceptors [38]. In eye imaginal discs overexpressing *fuss*, neither loss of Elav nor Chaoptin was detected (Fig 1C and 1D, arrow). This shows that cells overexpressing *fuss* still acquired a neuronal- as well as a photoreceptor fate. In the central nervous system (CNS), *fuss* is expressed in postmitotic interneurons during development, and its expression is maintained in adulthood [30]. Therefore, we focused in more detail on photoreceptor development. After acquiring photoreceptor fate, these cells start to protrude their axons into the larval optic lobe. Photoreceptors R1-R6 target the lamina neuropil (Fig 1C, arrow), whereas R7 and R8 axons migrate deeper into the medulla neuropil (Fig 1C, arrowhead). This leads to a very specific pattern, which can be observed in larval optic lobes of control larvae (Fig 1C), but upon *fuss* overexpression in developing photoreceptors, this pattern is strongly disturbed (Fig 1D, arrowhead).

### RNAseq reveals downregulation of the PAX2 homolog Shaven after *fuss* overexpression

**Fig 2:**
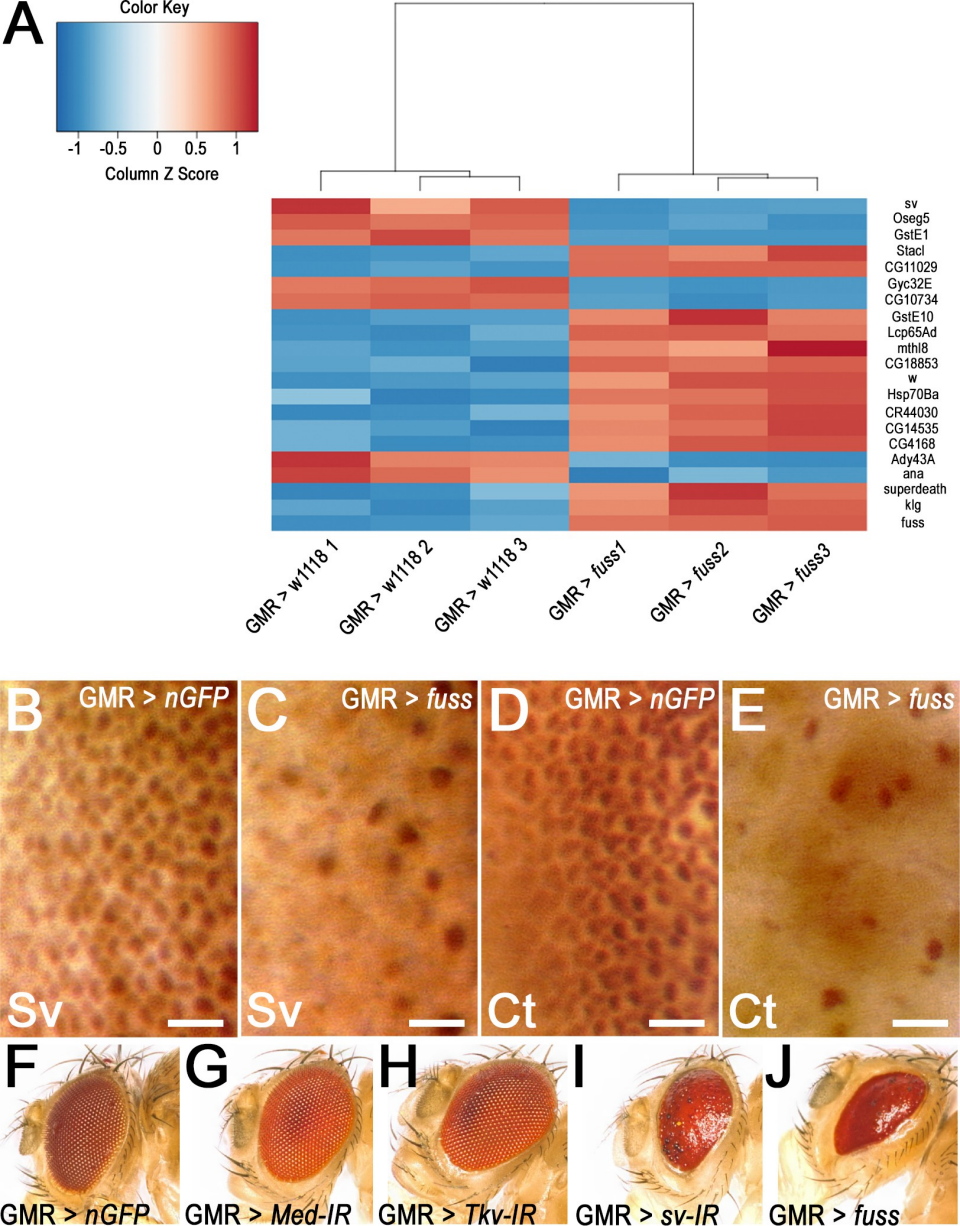
*fuss* overexpression leads to Ct and Sv downregulation and inhibits eye differentiation. (A) Heatmap depicts the top 21 dysregulated genes in Fuss overexpressing eye imaginal discs in contrast to control eye imaginal discs. (B) Shaven (Sv) expression in control eye discs. (C) Shaven (Sv) expression in Fuss overexpressing eye discs. (D) Cut (Ct) expression in control eye discs (E) Cut expression in Fuss overexpressing eye discs. (F) Adult eyes expressing UAS-*Stinger* with GMR-Gal4. (G) Adult eyes expressing UAS-*Med*-IR with GMR-Gal4. (H) Adult eyes expressing UAS-*Tkv*-IR with GMR-Gal4. (I) Adult eyes expressing UAS-*sv*-IR with GMR-Gal4. (J) Adult eyes expressing UAS-*fuss* with GMR-Gal4. DAB staining visualized by light microscopy, images were taken from the posterior side of the eye imaginal disc and the focus was set to the plane, where cone cell nuclei are localised (B-E). Scale bars: 10μm.

To achieve a better overview of genes and processes which become dysregulated by *fuss* overexpression in the larval eye imaginal disc, we extracted RNA from eye discs of late third instar larvae of controls (GMR-Gal4 > w1118) and experimental flies overexpressing *fuss* (GMR-Gal4 > UAS-*fuss*), reverse transcribed it and prepared NGS libraries, which then were sequenced. Comparing gene expression profiles of both genotypes, we found that Fuss was highly enriched in GMR-GAL4; UAS-*fuss* eye discs, which was a proof of principle that the experiment was successful (Fig 2A). Among 360 genes which showed an altered expression in contrast to controls with an adjusted *p*-value < 0.01, one gene, *shaven (sv)*, was especially interesting (S1 File). Sv, the Pax2 homologue in *Drosophila melanogaster*, is needed for the proper differentiation of cone, primary pigment and bristle cells and the loss of *sv* can result in the well-known glazed eye phenotype [39, 40]. This phenotype is strongly reminiscent of the phenotype observed of *fuss* overexpression with GMR-GAL4. Therefore, eye imaginal discs were stained with antibodies against Sv and its expression was indeed strongly reduced compared to controls (Fig 2B and 2C). Another protein, which is crucial for cone cell differentiation, is Cut (Ct), a homeobox containing transcription factor. Ct expression is induced by the transcription factor Sv [41] and therefore also *ct* expression is severely reduced upon *fuss* overexpression (Fig 2D and 2E). As mentioned before, Fuss can also inhibit Dpp signalling, although we found no altered expression of TGF-ß pathway components in our RNAseq data. In addition, we knocked down Medea (Fig 2G) and the type I receptor Tkv (Fig 2H) with the GMR-Gal4 driver line and did not find any defects in adult eyes compared to controls (Fig 2F). Besides that, we also knocked down *sv* (Fig 2I) and indeed, the phenotype is very similar to that of the *fuss* overexpression (Fig 2J). The eyes have a strong reduction in the number of lenses and show a smoother surface than controls. The phenotype of the sv knockdown is not as strong as the *fuss* overexpression, the reason for this might be, that the downregulation of sv in the knockdown is not as strong as after *fuss* overexpression or that additional processes are disrupted as there are 360 genes significantly altered in expression after *fuss* overexpression.

### Fuss overexpression results in loss of cell types and increased apoptosis

**Fig 3:**
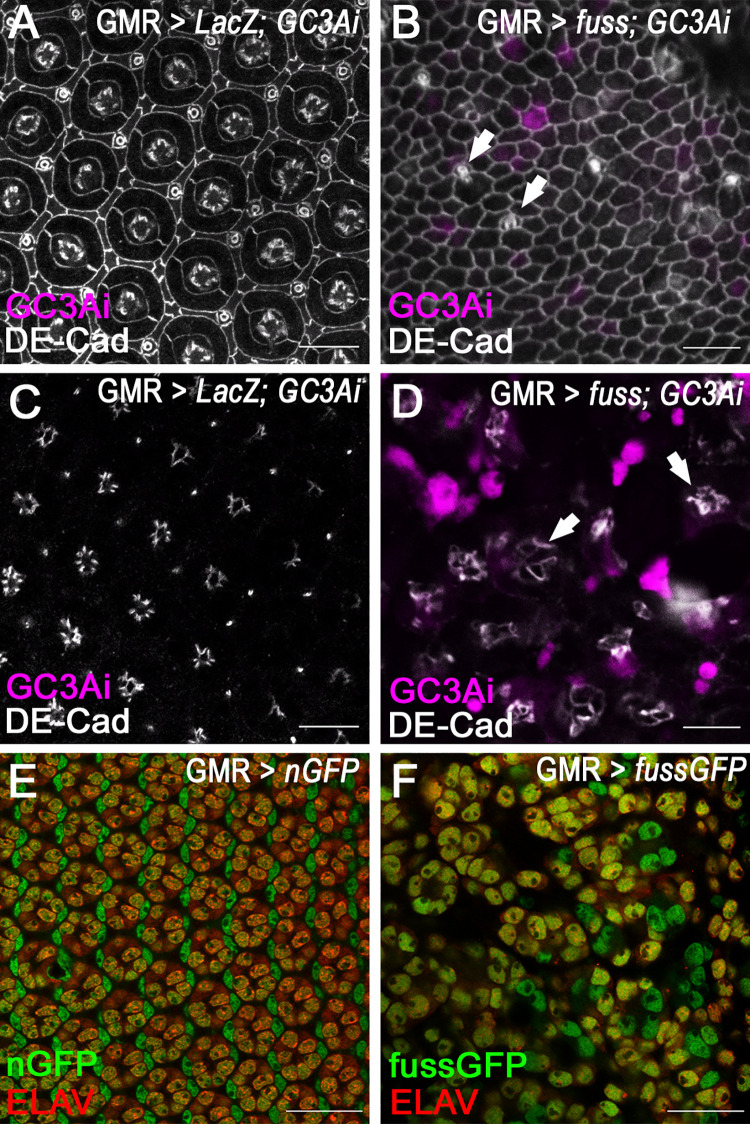
In *fuss* overexpressing pupal retinas cell organisation is completely lost and cell death is increased. (A) Organisation of cone cells, primary, secondary, tertiary and bristle cells in a pupal retina 40 hrs after puparium formation of control flies visualized with DE-CADHERIN (DE-CAD, gray) staining. No G3CAi (magenta) signal can be observed. (B) After *fuss* overexpression with GMR-Gal4 mainly one indefinable cell type differentiates and some single bristle cells are visible on the pupal retina surface (arrows) visualized with DE-CADHERIN (DE-CAD, gray) staining. G3CAi signal (magenta) is from cells directly below the retinal surface. (C) In a lower layer of the pupal retina photoreceptor axons are arranged circularly in controls and no G3CAi signal (magenta) can be observed. (D) After *fuss* overexpression circular arrangement of photoreceptor axons is strongly impaired (gray, arrows) and many apoptotic cells marked with G3CAi (magenta) signal can be observed. (E) In pupal retinas photoreceptor nuclei marked with ELAV expression (yellow) are arranged in a hexagonal array in controls (green). (F) In pupal eye discs of GMR-Gal4; UAS-*fussGFP* flies the ELAV positive photoreceptor nuclei are strongly disturbed in their patterning. Confocal images in A and B are stacks from three single focal planes. Residual confocal images are from single focal planes. Scale bars: 10μm.

To understand the developmental defects behind the smooth eye phenotype, pupal development of the overexpression eyes was analysed. In control eye imaginal discs of pupae (GMR-Gal4; UAS-*nGFP*), which have pupariated for 40 h, a highly ordered pattern of different cell types can already be observed. In the middle of an ommatidium, four cone cells, which secrete the lens, are surround by two primary pigment cells. The single ommatidia are separated from each other by secondary and tertiary pigment cells as well as bristle cells (Fig 3A). In pupal eye imaginal discs, where *fuss* was overexpressed via the GMR-GAL4 driver line (Fig 3B) most distinct cell types are lost on the surface of the pupal retina in contrast to controls, except for some single bristle cells which are visible (Fig 3B, arrows) but most of the cells which are present are of indefinable cell fate. However, these cells might develop to pigment cells, because the adults develop flat, structureless, but red eyes. With a fluorescent apoptosis sensor called GC3Ai [42], the *fuss* overexpressing retinas show an increase in apoptotic events in a deeper layer of the developing retina (Fig 3D) which could also account for the reduced size of the adult eye field if compared to the *sv* knockdown. As observed in larvae, the photoreceptor axons show a perturbed arrangement compared to controls (Fig 3D), but are still expressing the neuronal marker Elav (Fig 3F), although the positioning of photoreceptor nuclei is deranged. Additionally, large gaps can be observed between individual photoreceptor cell groups, which could be the result of apoptosis. (Fig 3F).

**Fig 4:**
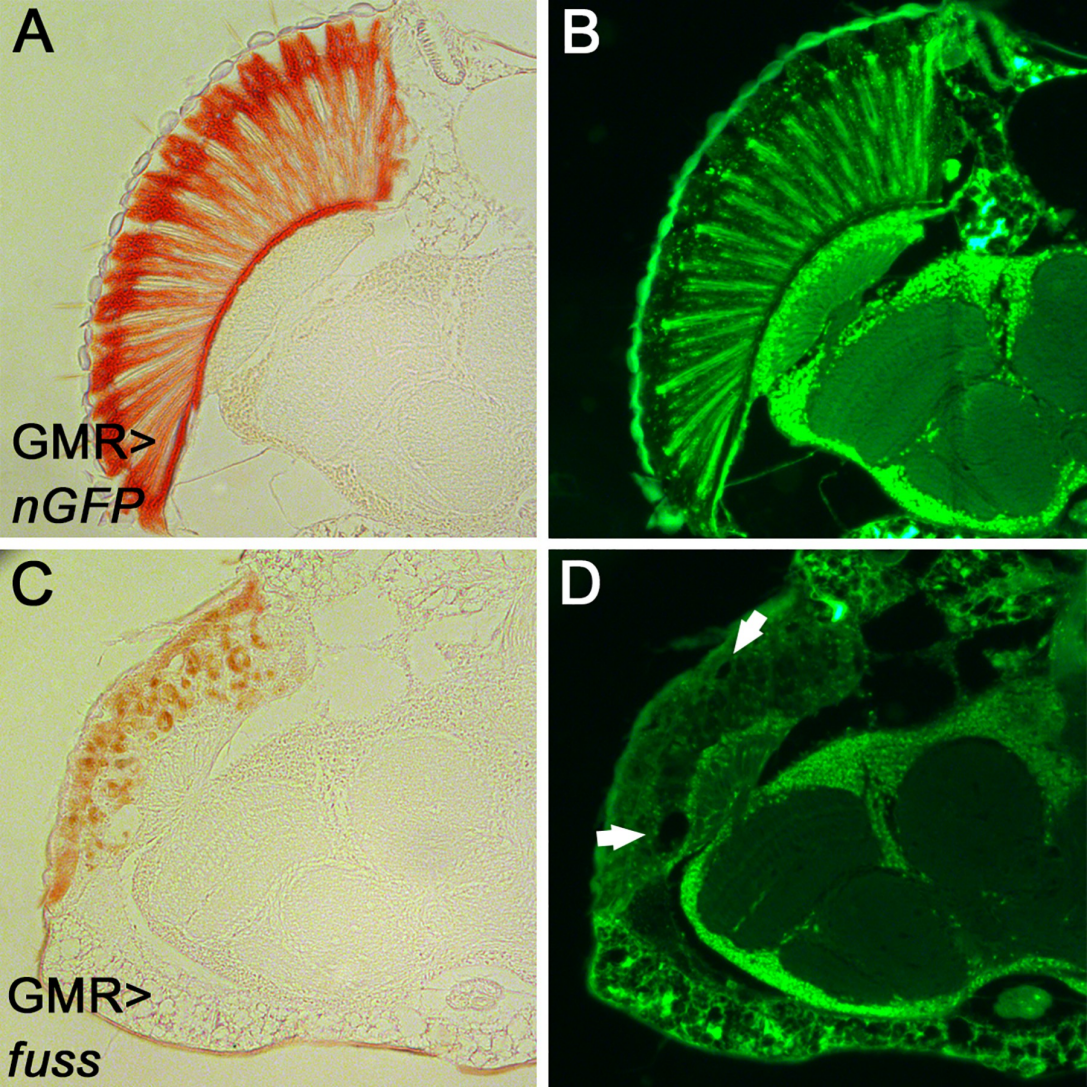
*fuss* overexpression during development disintegrates adult eye structure. (A) Paraffin sections of control heads illuminated with light. (B) Paraffin section of control heads illuminated with 470nm wavelength. (C) Paraffin section of a GMR-Gal4; UAS-*fuss* head illuminated with light shows small pigment granule containing cells. (D) Paraffin section of a GMR-Gal4; UAS-*fuss* head illuminated with 470nm wavelength reveals vacuoles (arrows), loss of adult eye structure including photoreceptors and reduced lamina size.

The final adult eye differentiation pattern was analysed by paraffin sections of heads of GMR-Gal4; UAS*-fuss* flies (Fig 4A and 4B) as well as controls (GMR-Gal4; UAS-*nGFP*). In *fuss* overexpressing eyes (Fig 4C and 4D) the photoreceptors and their rhabdomeres, which can be observed by a bright fluorescent signal in the controls (Fig 4A) are completely lost. Although they were determined to become photoreceptors as observed in Fig 1D, they are probably removed by apoptosis (Fig 3D) during pupal development, which also affects the integrity of the adult lamina. Furthermore, vacuoles can be observed (Fig 4D, arrows) which are probably also the outcome of the cell death observed during pupal stage (Fig 3D). These sections show, that the indefinable cells observed in pupal retinas have a completely different shape than those of secondary or tertiary pigment cells in controls (Fig 4A), but still contain pigment granules (Fig 4C). Thus, these cells rather adopt a basal pigment cell fate but don´t acquire the correct shape.

Obviously, overexpressing *fuss* in differentiating cells posterior to the morphogenetic furrow highly impacts their specification. Photoreceptors are determined but are abolished during development and residual cells are prevented to adopt their natural fate and nearly all are transformed to a basal pigment cell fate. Therefore, *fuss* overexpression is able to completely inhibit differentiation but, in this context, no striking increase in proliferation was observed.

### Early induction of *fuss* overexpressing clones inhibits eye differentiation and leads to eye disc outgrowths

The ability of Ski/Sno proteins to function as proto-oncogenes is often linked to their capacity to inhibit the antiproliferative effects of TGF-ß signalling. To check for early proliferative defects, we decided to induce *fuss* overexpression clones in first instar larvae. 48 hours after egg laying, we heat-shocked the first instar larvae with the genotype (P{hsp70-flp}1/+; Fuss-GFP/+; GAL4-Act5C(FRT.CD2).P) for 12 minutes to induce *fuss* overexpressing clones. Late third instar larvae were dissected, and tissue was analysed for *fuss* overexpressing clones and their effects on development.

**Fig 5:**
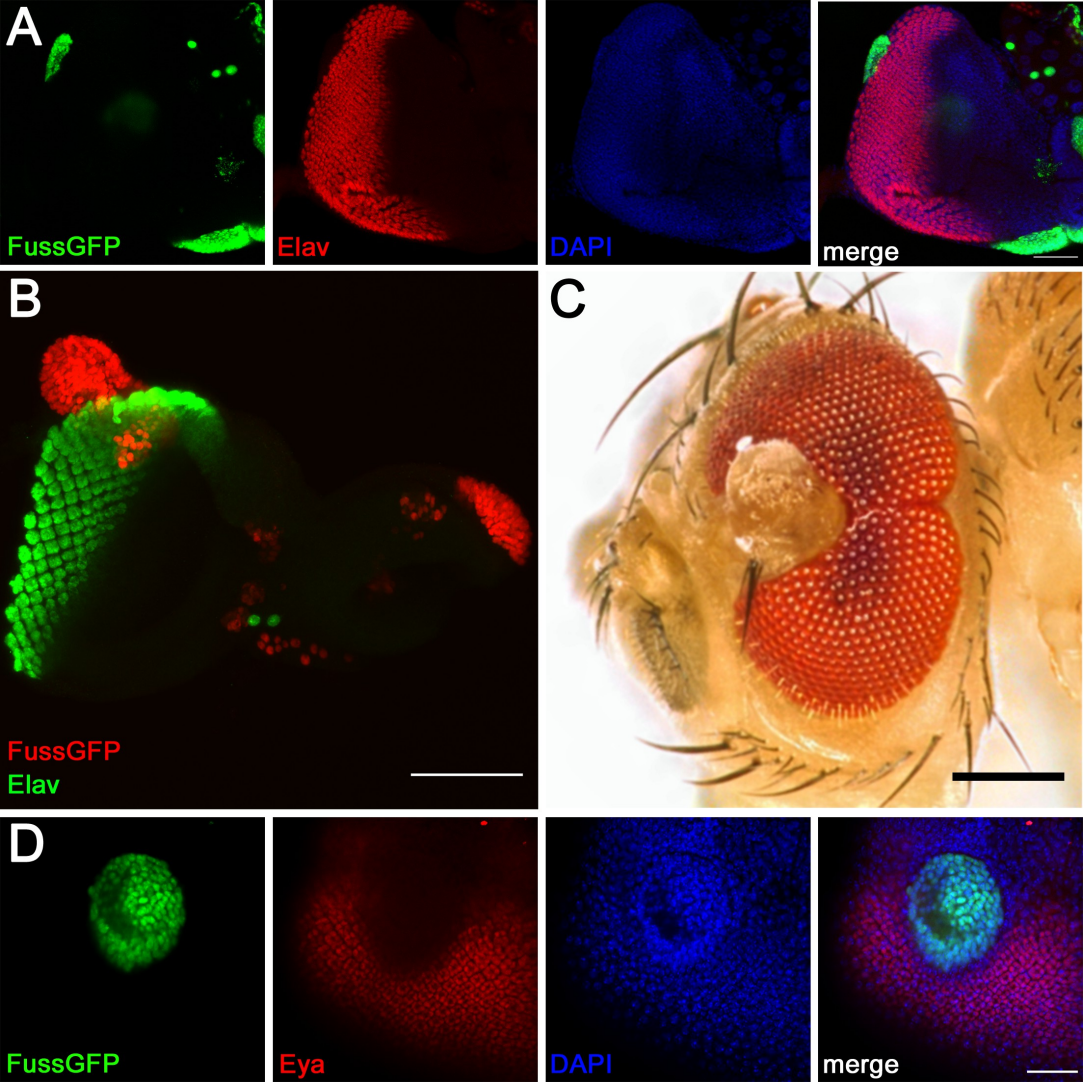
*fuss* overexpressing clones lead to undifferentiated outgrowths from eye imaginal discs. (A) *fuss* overexpressing clones (green) in eye imaginal discs are not expressing the late neuron differentiation marker Elav (red). DAPI (blue) (B) *fuss* overexpressing clones (red) extensively outgrow from eye imaginal discs. Elav (green). (C) Adult survivors exhibit large outgrowths from eyes. (D) *fuss* overexpressing clones (green) in larval eye imaginal discs are not expressing the early differentiation marker Eyes absent (Eya, red). DAPI (blue). All images are stacks. Scale bars: 50 μm (A, B), 25 μm (D).

Surprisingly, as shown in Fig 5, we detected a completely different behaviour of *fuss* overexpression. *fuss* overexpressing clones in eye imaginal discs result in tissue outgrowths. To check the cellular identity of these outgrowing cells, we first tested for the neuronal differentiation marker Elav, which would be expected in photoreceptor cells. However, these outgrowths lack the late differentiation marker Elav completely and can form big bulbous structures which protrude from the eye imaginal disc (Fig 5A and 5B). All heat shocked larvae die at the latest during pupal stages, therefore we decreased the time of the heat shock, which enabled us to obtain single adult survivors. Some of these survivors had undifferentiated tissue growing out from their eye, which apparently was generated by *fuss* overexpressing clones (Fig 5C). To further understand the *fuss* overexpression defects, we were searching for early retinal differentiation markers. Dpp signalling is assumed to be required for the expression of early retinal differentiation markers *sine oculis*, *dachshund* and *eyes absent* (*eya*) [43, 44]. Previous studies have shown, that overexpression of *fuss* can interfere with Dpp signalling [4] and we could indeed confirm that *fuss* overexpressing clones lack *eya* expression (Fig 5D) and therefore, Fuss might inhibit Dpp signaling in the eye imaginal disc as well. All in all, initiation of photoreceptor differentiation is already impaired in *fuss* overexpressing clones.

### *fuss* overexpressing clones exhibit increased proliferation

**Fig 6:**
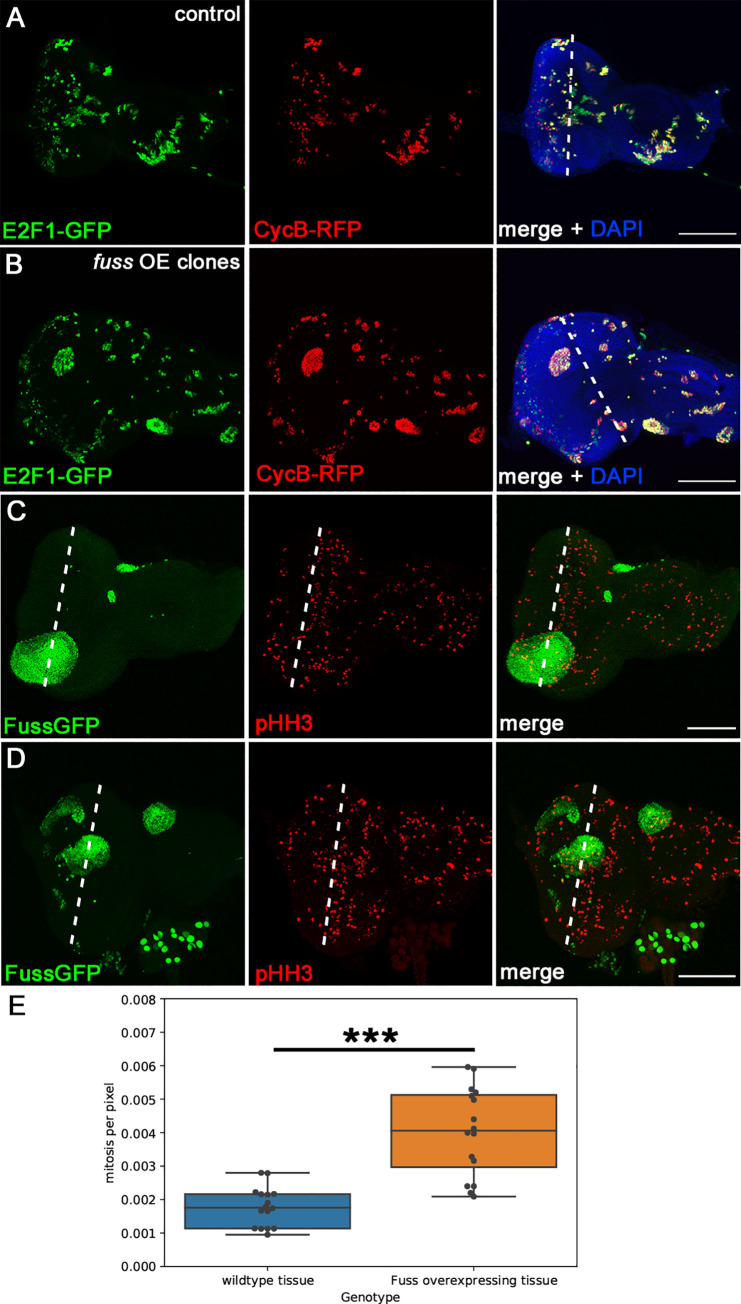
*fuss* overexpressing clones exhibit increased proliferation in the eye imaginal disc. (A) Eye imaginal discs with clones expressing the Fly-FUCCI system. E2F1 is fused to GFP (green) and CycB is fused to RFP (red). Dashed line marks the morphogenetic furrow. (B) Eye imaginal discs with clones expressing the Fly-FUCCI system and Fuss (green). Dashed line the marks morphogenetic furrow. (C, D) FussGFP overexpressing clones (green) and mitotic cells (pHH3, red) Dashed line marks the second mitotic wave. (E) Wildtype tissue (0.001783, n = 16) exhibits less mitotic events per pixel than *fuss* overexpressing tissue (0.004029, n = 16). Two-sided Mann-Whitney U test was used to calculate p-value. ***p<0.001. All images are stacks. Scale bars: 100μm.

However, this does not explain, why *fuss* overexpressing clones lead to tissue outgrowths from the eye. Generally, if eye development is inhibited, this would lead to a transformation from eye to head capsule tissue only and not to additional head capsule tissue as observed [45]. In contrast to *eya* mutant eye discs, where proliferation is strongly reduced [46], the *fuss* overexpressing clones seem to show increased proliferation. To further analyse cell proliferation, we used the Fly-FUCCI system, where degrons of E2F1 and CycB have been fused to GFP and mRFP, respectively, to visualize the cell cycle behaviour of cells [47]. Cells from anaphase to the G1 to S transition are green, S phase cells are red, and cells in G2 and early mitosis are yellow. Expressing the Fly-FUCCI system via the Flipase technique in eye imaginal discs (Fig 6A), we found that many cells posterior to the morphogenetic furrow are green or red, therefore in G1 or S-Phase and only some where yellow, thus in early mitosis. But when we coexpressed *fuss* with the Fly-FUCCI system many more cells where yellow posterior to the morphogenetic furrow and consequently where in G2-Phase or undergoing mitosis (Fig 6B). Interestingly, the shape of *fuss* overexpressing clones were highly different to that of control clones. Whereas control clones integrate into the patterning of the developing eye imaginal discs, *fuss* overexpressing clones might not react to Dpp signalling as shown before, have a rather elliptical shape and are protruding from the eye imaginal disc. Because of this experiment we expected, that *fuss* overexpressing clones have a higher division rate than controls. We chose to use an antibody against phosphorylated Histone H3, a marker for mitosis, to specifically identify mitotic cells and count them. In this assay we only counted cells, behind the second mitotic wave (smw), because after the smw cells start to differentiate instead to proliferate. As controls we used tissue which was not overexpressing *fuss*, therefore we counted the number of pHH3 positive cells inside *fuss* overexpressing clones and outside of it, and measured the area in pixels of the *fuss* overexpressing clonal tissue and the wildtype tissue and divided the number of mitotic cells by the amount of pixels. We found significantly more pHH3 positive cells per pixel in the *fuss* overexpressing clones than in the surrounding wild type tissue (Fig 6C and 6D, S2 File), demonstrating that *fuss* overexpression leads to increased proliferation in developing eye imaginal discs in contrast to controls (Fig 6E).

### *wingless* is expressed in *fuss* clonal outgrowths

**Fig 7:**
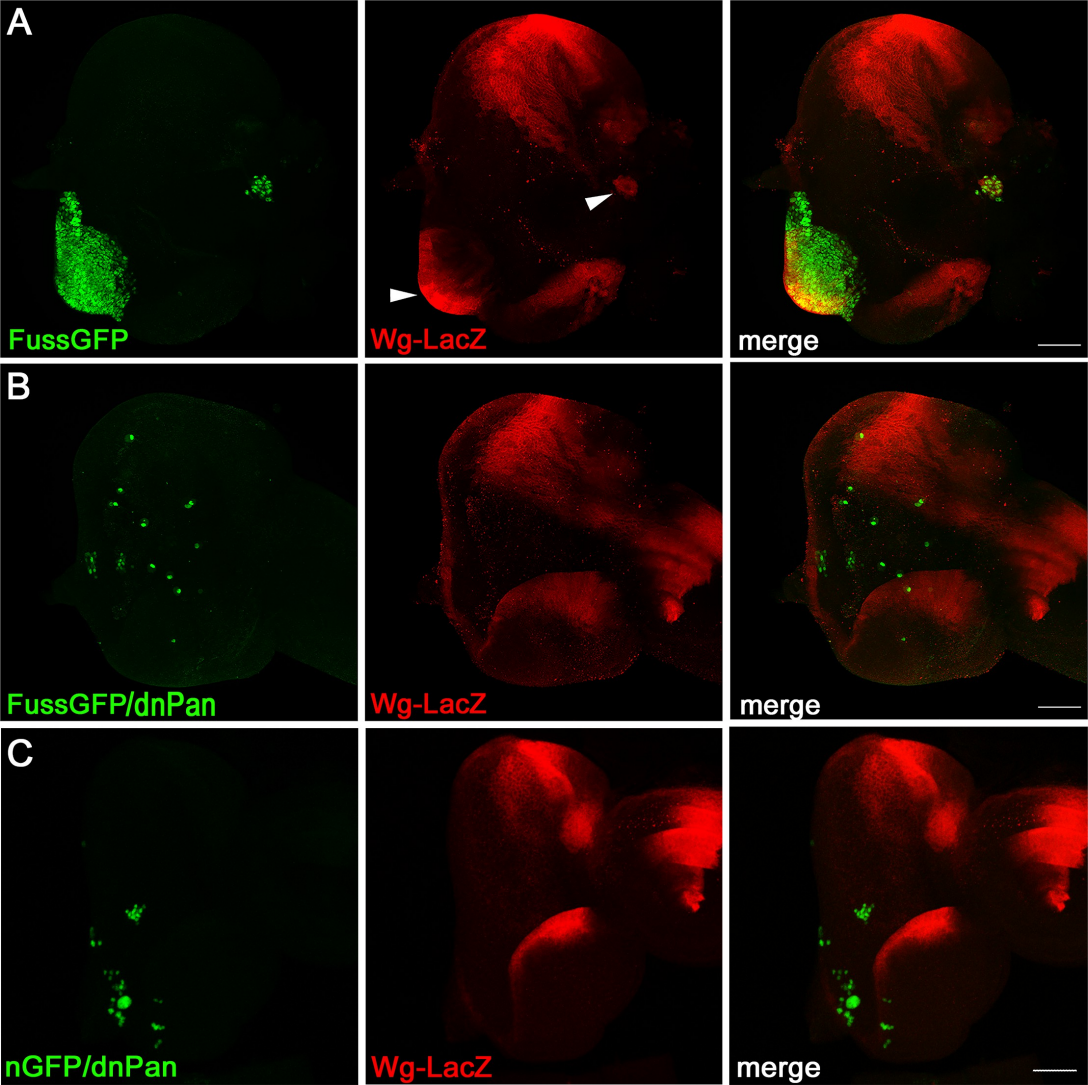
*fuss* overexpressing clones exhibit *wg* expression. (A) FussGFP overexpressing clones (green) show LacZ expression (red) under the control of a *wg* promotor. (B) Simultaneous expression of FussGFP and dnPan (green) inhibits LacZ expression (red) under the control of a *wg* promotor and clonal tissue is strongly reduced in size. (C) dnPAN expressed together with nuclear GFP (green) show no expression of LacZ under the control of a wg promotor (red). All images are stacks. Scale bars: 50 μm.

Besides eye differentiation, Dpp signalling is also required to inhibit *wg* expression in the eye imaginal discs [36]. In eye imaginal discs of third instar larvae, *wg* expression can normally only be observed at the dorsal and ventral margins of the eye disc, where it is supposed to promote head capsule structures instead of eye tissue [48], but in earlier stages *wg* is expressed in the whole prospective eye [49] and this is around the time we induce the *fuss* overexpressing clones. Loss of Dpp signalling leads to overgrowth and ectopic *wg* expression in the eye imaginal disc [36] and loss of *eya* expression might not only be the result of *fuss* dependent inhibition of Dpp signalling, but also ongoing Wg signalling, as it has been shown by ectopic Wingless signalling clones [50]. To test if *fuss* overexpression clones continue expressing *wg* from earlier stages on via a possible inhibition of Dpp signalling, we used a wg-LacZ reporter construct to visualise *wg* promotor activity in the eye imaginal disc and as shown in Fig 7A *fuss* overexpressing clones exhibit indeed LacZ expression supporting the hypothesis that the inhibition of Dpp signalling allows continuous *wg* expression in these cells. Wingless signalling is also involved in promoting the proliferation of cells anterior to the morphogenetic furrow and ectopic Wingless signalling leads to increased proliferation [51]. One possible hypothesis for the overproliferation observed with *fuss* overexpressing clones might be that Fuss acts again as an inhibitor of Dpp signalling in the eye imaginal disc allowing continuous *wg* expression, which might lead to the excess proliferation in the eye imaginal disc. To test this hypothesis, we tried to generate clones, which besides *fuss*, also express a knockdown construct for *wg*, but this approach turned out to be highly lethal. In a second approach, we generated clones which expressed *fuss* and a dominant negative form of *Pangolin* (*dnPan*), because the effects of *wg* overexpression can be suppressed by dominant negative Pangolin, even when expressed from the same cell [52] and it has been shown, that *wg* expression can be autoregulated endogenously [53, 54] or ectopically in some tissues [55]. *fuss/dnPan* overexpressing clones (Fig 7B) were not only consistently smaller compared to *fuss* overexpressing clones (Fig 7A), but the clonal tissue did also not outgrow anymore. *fuss/dnPan* overexpressing clones did not exhibit *wg* expression anymore, as observed with the absence of LacZ staining, supporting our hypothesis that Fuss, via inhibition of Dpp signaling, might be able to allow *wg* expression from earlier stages to continue and *wg* expression and Wg signalling in these clones might promote the outgrowths from the eye disc. Similarly, overexpressing dominant negative Pan together with nuclear GFP lead to small clones and *wg* expression was also not increased (Fig 7C).

## Discussion

In this work, we addressed the question if Skor/Fuss proteins, members of the Ski/Sno family, retained the function of Ski and Sno to induce uncontrolled proliferation as observed in early stages of tumorigenesis.

First, the overexpression of *fuss* posterior to the morphogenetic furrow with the GMR-Gal4 driver line resulted in a nearly complete loss of all cell types in the adult eye. During development, photoreceptor axons were not able to target the appropriate layers of the optic lobe anymore and cone cells, primary pigment cells and bristle cells were transformed into a basal pigment cell fate. This transformation was caused by the inhibition of *sv* expression, which is crucial for accessory cell differentiation. Additionally, increased apoptosis during pupal development lead to the removal of photoreceptors and lastly adult eyes only consisted of cells containing pigment granules. This lack of differentiation cannot be explained by the Dpp inhibiting role Fuss exerts, when overexpressed [4], because inhibiting the Dpp signaling pathway via knockdown of Tkv or Med had no effect. Photoreceptor axon guidance is impaired, if Dpp signaling is disrupted in photoreceptors by the expression of the inhibitory Smad Dad [56]. Thus, the observed photoreceptor axon guidance phenotype, when *fuss* is overexpressed with GMR, could indeed be a result of Dpp signaling inhibition. However, the loss of nearly all eye cell types is due to other effects (e.g. downregulation of *sv* and apoptosis) than Dpp signaling repression alone, because loss of Dpp signaling behind the morphogenetic furrow only results in mild patterning defects of the pupal retina [57]. Nonetheless, the inhibition of cell differentiation has already been shown in other cancer models e.g., when two copies of the constitutive active form of the receptor tyrosine kinase dRET^MEN2B^ are expressed with the GMR-Gal4 line, pupal retinas are devoid of any distinguishable cell types [33]. This phenotype is indistinguishable from the phenotype of the pupal retinas generated by the overexpression of *fuss* via GMR-Gal4. In a screen for novel oncogenes from breast cancer patients, human transgenes have been overexpressed with the GMR-Gal4 driver line. Overexpression of human RPS12, a subunit of the small ribosomal subunit, whose expression is increased in various cancer types, leads also to a glazed eye phenotype [58]. Therefore, different oncogenes can result in different outcomes when expressed with the GMR-Gal4 driver line and are not always leading to massive tissue overgrowth like the Yorkie overexpression [59]. Most importantly, with this approach to overexpress *fuss* in cells which already were destined for acquiring a cell fate and have left the cell cycle, we were not able to induce increased proliferation anymore, but could prevent cell differentiation.

Consequently, we switched to a more pluripotent cell type in the eye imaginal disc [60] and induced *fuss* overexpressing clones prior to the formation of the morphogenetic furrow. These results let us assume that in this context, *fuss* overexpressing clones do not react to the antiproliferative effects of the Dpp morphogen anymore. Instead, *wg* expression and thus, proliferation promotion might be maintained. This leads to outgrowths of clonal tissue from the eye imaginal disc of third instar larvae, which showed an increased number of mitotic events. If these flies survived to adulthood, undifferentiated, extra tissue was visible in the complex eye.

An analogous mechanism can be observed in tumors which overexpress Ski or Sno. The TGF-ß signaling pathway also acts anti-proliferative, but this action is inhibited by the increased presence of Ski/Sno proteins. Therefore, the molecular mode of action is similar to the human Ski/Sno proteins. The function of Ski and Sno is highly context dependent, as they can fulfill an anti-oncogenic or pro-oncogenic role depending on the cancer type or status of the cancer. We also observed this with *fuss* overexpressing clones. Only when induced 48h after egg laying, we found additional tissue in late third instar larvae and only in eye imaginal discs, because here, Dpp counteracts the proliferative effects of Wg signaling. When *fuss* is overexpressed in the wing disc or after induction of the morphogenetic furrow differentiation is inhibited, this results in a wing with truncated veins [4] or in a smooth eye surface (this work). This is also underlined by RNAseq data from eye and wing imaginal discs, where *fuss* was overexpressed with the GMR-Gal4 and Nub-Gal4 driver line, respectively. In the eye dataset, *wg* expression in eye imaginal discs is not significantly different from control eye discs, whereas *wg* expression in *fuss* overexpression wing discs is significantly reduced in contrast to control wing discs (S1 Fig).

Thus, we were able to show that the Skor protein Fuss in *Drosophila melanogaster* still retained the function of the Ski/Sno proteins by inhibiting differentiation but inducing hyperproliferation. But the hallmarks of real tumorigenesis are lacking, because at some point during pupal development, proliferation stops, and these cells become protruding head tissue as it could be observed in complex eyes of surviving flies. Furthermore, there was no evidence of an epithelial-mesenchymal transition because *fuss* overexpressing clones maintained their epithelial fate. It will be of high interest if future studies can find similar results in overexpression studies for the vertebrate Skor proteins or detect increased expression of these proteins in specific cancer types.

## Material and methods

### Fly husbandry and stocks

Flies were raised at 25°C under a 12 hr/12 hr light/dark cycle. Fly lines obtained from the Bloomington Drosophila Stock Center were: P{GAL4-ninaE.GMR}12 (BDSC #1104), w1118; P{UAS-Stinger}2 (BDSC #84277), w1118; snaSco/CyO, P{en1}wgen11 (BDSC #1672), y1 w1118; P{UAS-pan.dTCFΔN}4 (BDSC #4784), y[1] w[*]; P{w[+mC] = GAL4-Act5C(FRT.CD2).P}S (BDSC #4780), P{ry[+t7.2] = hsFLP}1, y[1] w[1118]; Dr[Mio]/TM3, ry[*] Sb[1] (BDSC #7), P{AyGAL4}25 (BDSC #4412), w1118 (BDSC #3605), y1 v1; P{TRiP.HMS05834}attP2 (BDSC #67973), y1 sc* v1 sev21; P{TRiP.GL01313}attP40 (BDSC #43961), y1 sc* v1 sev21; P{TRiP.HMS04501}attP40 (BDSC #57303), w[1118]; Kr[If-1]/CyO, P{ry[+t7.2] = en1}wg[en11]; P{w[+mC] = UAS-GFP.E2f1.1–230}26 P{w[+mC] = UAS-mRFP1.NLS.CycB.1-266}17/TM6B (BDSC #55122), Tb[1], w*; KrIf-1/CyO; P{UAS-GC3Ai}3 (BDSC #84343). Additionally, Nub-Gal4 (J.F. de Celis, Madrid) was employed.

### Immunohistochemistry

For analysis of *fuss* overexpressing clones 48 hrs after egg laying, the larvae were heat shocked at 37°C for 12 min. Then, for all experiments late third-instar larvae were used for dissection and immunohistochemistry. By pulling the mouth hooks, the anterior mouth part including the eye imaginal discs still attached to the brain were removed from the rest of the larva and then fixed by incubation in 4% PFA in PBS for 20 min. The specimen was washed three times with PBST (PBS with 0.1% Triton-X) for 20 min and incubated in PBST supplemented with 5% normal goat serum and primary antibodies over night at 4°C. The specimen was washed three times with PBST for 20 min and incubated in PBST supplemented with 5% normal goat serum and secondary antibodies over night at 4°C. The specimen was washed once with PBST for 20 min, then incubated in PBST supplemented with 1 mg/ml 4′,6-Diamidin-2-phenylindol (DAPI) for 20 min and washed three times with PBST for 20 min. The eye imaginal discs were dissected and mounted using VECTASHIELD Antifade Mounting Medium (Vector Laboratories). Developmental studies Hybridoma Bank (DSHB) antibodies were: LacZ (JIE7, 1:50), Eyes absent (eya10H6, 1:50), Elav (Rat-Elav-7E8A10, 1:50), Cut (2B10, 1:20), Chaoptin (24B10, 1:50). Additional antibodies were: Sv/Pax2 (1:100, gift from Markus Noll), GFP (rabbit 1:1000, ThermoFisher), pHH3 (rabbit 1:2000, Cell signaling technology). Secondary antibodies were used with a dilution 1:200 overnight at 4°C. Secondary antibodies were goat anti-mouse, anti-rabbit, anti-rat and anti-guinea pig Alexa Fluor 488, 555 and 594 (ThermoFisher). For anti-Cut and anti-Sv stainings we used the Anti-Mouse / anti-Rabbit HRP-DAB IHC kit (abcam) to increase sensitivity and reduce background.

### Generation of FLP-out clones

In general, we crossed virgins carrying the P{hsp70-flp}1 allele homozygously to males carrying the GAL4-Act5C(FRT.CD2).P allele. Before the cross was placed for 2h on standard food supplemented with dry yeast at 25°C, flies were allowed to mate for at least three days. The adult flies were removed from the vial and progeny was allowed to develop for 46h at 25°C. Progeny was heatshocked for 12 minutes at 37°C and placed again at 25°C. Late third instar larvae were then dissected.

### Retina dissection and immunostaining

White pupae were collected and aged at 25°C for 40 hrs. The brains with the attached eye discs were dissected in PBS and placed in PBS with 4% PFA on ice until all brains from one genotype were dissected. Afterwards the brains were fixed for another 20 min with 4% PFA in PBS at room temperature. The brains with the attached eye discs were stained with rat-anti-DE-cadherin (DCAD2, 1:50, DSHB) or rat-anti-ELAV (Rat-Elav-7E8A10, 1:50) and goat-anti-rat Alexa Fluor 555 (1:200, ThermoFisher) in PBST 0.1% with 5% normal goat serum (NGS). After staining, the eye discs were removed from the brains directly on the mounting slide in a drop of PBST 0.1% and mounted using VECTASHIELD Antifade Mounting Medium (Vector Laboratories).

### Quantification of pHH3 positive cells

Only eye imaginal discs where the second mitotic wave was clearly detectable via pHH3 staining were used. Mitotic cells in *fuss* overexpressing clones and in wildtype tissue were counted. The area of *fuss* overexpressing clones and wildtype tissue was measured with the measurement tool of ImageJ. The number of mitotic cells inside a *fuss* overexpressing clone was divided by its area and the number of mitotic cells inside the wildtype tissue was divided by the wildtype tissue´s area. The acquired data was visualized with Python and the Matplotlib and Seaborn libraries. Statistics were calculated with the SciPy library.

### Paraffin sections

Paraffin sections were performed from two-day old adult flies. Flies were fixed with carnoy (ethanol:chloroform:acetic acid at a proportion 6:3:1), dehydrated in ethanol, and embedded in paraffin. Paraffin sections (7 μm) from 10 flies of each genotype were analysed under a fluorescence microscope.

### RNA extraction, library generation and sequencing

Per replicate and genotype 40 eye antennal discs or 30 wing discs from third instar larvae were dissected. RNA was extracted via peqGold MicroSpin Total RNA Kit. Library preparation and RNA-Seq were carried out according to the NEBNext Ultra RNA Library Prep protocol, the Illumina HiSeq 1000 System User Guide, and the KAPA Library Quantification Kit—Illumina/ABI Prism User Guide. Library preparation and RNA-Seq were performed at the Genomics Core Facility “KFB—Center of Excellence for Fluorescent Bioanalytics” (University of Regensburg, Regensburg, Germany).

### RNA-Seq analysis

The reads were quantified with the R package Salmon [61] using the release of the *Drosophila melanogaster* genome BDGP6.22. The data was imported using tximeta [62] and analysed with DESeq2 [63]. Cut-off for significantly dysregulated genes was set with an adjusted *p*-value < 0.01. Top 21 differentially expressed genes between control and overexpression replicates were visualized with the heatmap.2 package in R.

## Supporting information

S1 Fig Wg is context dependent affected by Fuss overexpression. In wing disc *fuss* overexpression (Nub-Gal4 > UAS-*fuss*) leads to decreased *wg* expression in contrast to controls (Nub-Gal4 > w1118). In eye discs *wg* expression is unaffected by the overexpression of *fuss* (GMR-Gal4 > UAS-*fuss*) if compared to controls (GMR-Gal4 > w1118).
(TIF)

S1 File Differential expression analysis of *fuss* overexpressing eye discs and control eye discs. (XLSX)

S2 File Data for box plot. (XLSX)
