# Supplementary figures and images for: The Drosophila functional Smad suppressing element fuss, a homologue of the human Skor genes, retains pro-oncogenic properties of the Ski/Sno family
Source: PLoS One. 2022 Jan 14;17(1):e0262360. doi: 10.1371/journal.pone.0262360 (PMC8759651; doi:10.1371/journal.pone.0262360)

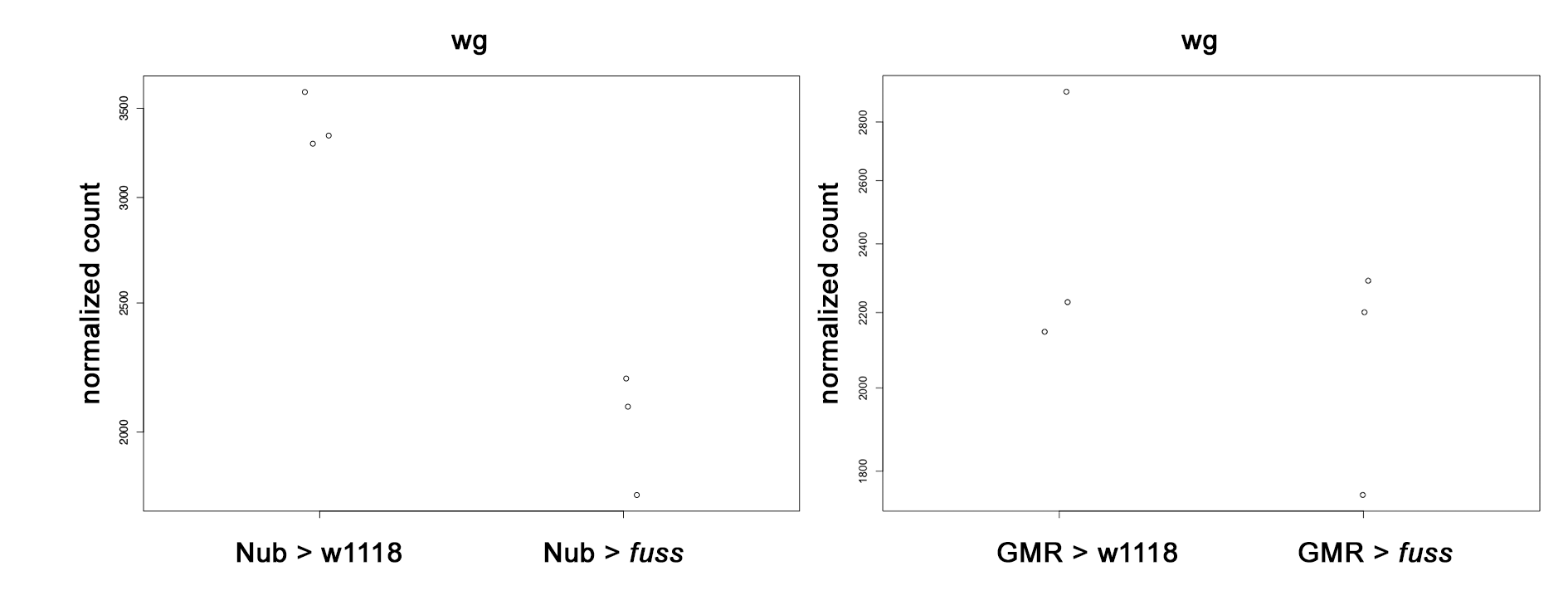

Supplement: S1 Fig — In wing disc fuss overexpression (Nub-Gal4 > UAS-fuss) leads to decreased wg expression in contrast to controls (Nub-Gal4 > w1118). In eye discs wg expression is unaffected by the overexpression of fuss (GMR-Gal4 > UAS-fuss) if compared to controls (GMR-Gal4 > w1118). (TIF) [file pone.0262360.s001.tif]
